# A Case of Migraine Aura Status in a Migraine Patient With Typical Aura Without Headache

**DOI:** 10.7759/cureus.50062

**Published:** 2023-12-06

**Authors:** Yasutaka Sadamoto

**Affiliations:** 1 Headache Center (Neurosurgery), Takanoko Hospital, Matsuyama, JPN

**Keywords:** visual aura, aura, migraine aura status, migraine with aura, migraine disorder

## Abstract

Migraine aura status (MAS) is a rare complication of migraine and is listed in the appendix of the International Classification of Headache, Third Edition. MAS occurs in migraine with aura (MwA) patients, with at least three auras occurring over a period of three days. We describe a case of MAS associated with a patient who had a migraine with a typical aura without headache (TAWH). She has experienced only visual auras, including periods of MAS complications, and has not experienced headaches or other auras, including sensory, speech, and motor.

The patient is a 45-year-old woman. She had discomfort with visual aura without a headache every two to three months. The visual aura began to gradually darken a portion of the right side of the visual field binocularly, which gradually expanded around it and was surrounded by iridescent flashes running in a zig-zag pattern to the periphery. The area of the visual field abnormality gradually decreased and disappeared after approximately 15 minutes. One day, she had an aura once after dinner, and when she awoke without any trigger after going to bed, the aura appeared and repeated approximately three times. She experienced three to four auras the next day and the day after. In the six-month follow-up period, there was no further recurrence of MAS.

Most previous reports of MAS have been associated with typical MwA, and there have been few other reports of MAS associated with TAWH as far as we could investigate.

## Introduction

Migraine aura status (MAS) is a rare complication of migraines and is listed in the Appendix of the International Classification of Headache, Third Edition (2018) [1]. The diagnostic criteria include a migraine fulfilling the criteria for 1.2 migraine with aura (MwA) or one of its subtypes and at least three auras occur over a period of three days [1]. It is commented that other neurological disorders, including reversible cerebral vasoconstriction syndrome (RCVS) and reversible posterior leukoencephalopathy syndrome (PRES), should be excluded by appropriate investigation. MAS has been reported since 1982, and the frequency of aura onset and number of days of continuous onset have been moderated from ICHD-II (2004) [2] to ICHD-III [1]. In a retrospective analysis of 477 cases of recurrent MwA, 1.7-4.2% of patients had MAS (includes proposed criteria) [3], patients with MAS were older at onset and had predominantly visual symptoms, normal neuroimaging results, and no sequelae compared to those with a typical aura [3]. However, three cases of MAS secondary to an organic brain lesion were reported [4].

In the literature, MwA complicated by MAS is often typical MwA. Herein, we describe a complicated case of MAS in a patient with only a typical aura without headache (TAWH).

## Case presentation

The patient was a 45-year-old woman who was diagnosed with mild hypertension during her medical checkup last year and was prescribed antihypertensive medication (calcium blocker amlodipine 2.5 mg/day) by her local physician. There was no other medical or family history including head injuries. She had no habit of drinking alcohol or smoking. She has a son who lives far away. She had discomfort with a visual aura without a headache every two to three months since she was approximately 35 years old. The visual aura began to gradually darken a portion of the right side of the visual field binocularly, which gradually expanded around it, and was surrounded by iridescent flashes running in a zigzag pattern to the periphery. The area of the visual field abnormality gradually decreased and disappeared after approximately 15 minutes. She was examined by a local ophthalmologist and was followed up without any abnormal findings, including a fundus examination. She did not experience headaches or sensory, speech, and motor auras after the visual aura. She experienced an aura at the end of March 2023. In May of the same year, her son’s health deteriorated. Consequently, she repeatedly traveled long distances by driving a car and was sleep-deprived.

On May 26 of the same year, she experienced an aura once after dinner, and when she awoke without any trigger after going to bed, the aura appeared and recurred approximately three times. On May 27, she experienced an aura once after breakfast and three times between evening and bedtime. On May 28, an aura appeared once after breakfast and twice at approximately 4:00 p.m. All auras were the same as usual.

On May 29, she visited our department for the first time. The patient’s blood pressure was 134/78 mmHg, pulse was 73/min, and temperature was 36.4 ℃, and her weight did not change during the year prior to the visit. She had clear consciousness, no muscle weakness in the extremities, normal deep tendon reflexes, no abnormalities in the cranial nervous system, no pyramidal or cerebellar signs, no complaints of numbness, normal conversation, normal mental status, and a normal fundus examination. Blood tests showed that her liver and kidney function parameters and blood glucose levels were within the normal ranges, with no inflammatory reaction or anemia. We considered it necessary to exclude organic brain diseases such as cerebrovascular disorders and epilepsy.

**Figure 1:**
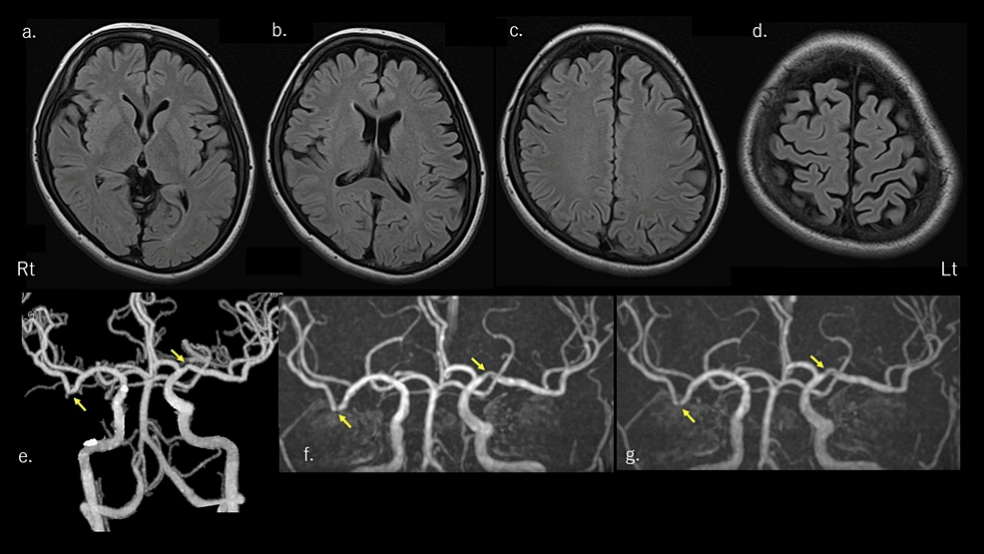
Brain magnetic resonance imaging (MRI), computed tomography angiography (CTA), and magnetic resonance angiography (MRA) The upper row images show brain MRI fluid-attenuated inversion recovery images performed at the time of initial diagnosis (day 3 of onset). (a-d): There were no abnormal findings. The lower row images show head CTA at the initial visit (day three onset) (e) and head MRA at days 35 and 84 onset (f and g). (e): There was no obvious stenosis in the main trunk artery, but there was slight stenosis in the left middle cerebral artery (MCA) M1 and right MCA M2 segment. (f, g): No obvious changes were observed on head MRA at 35 and 84 days of onset. Both had slight stenosis of the left MCA M1 and right MCA M2 segments. This was thought to be due to flexure or mild sclerotic changes.

The patient had mild claustrophobia and difficulty with prolonged magnetic resonance imaging (MRI). Unfortunately, only diffusion-weighted imaging (DWI), fluid-attenuated inversion recovery (FLAIR) sequences, and magnetic resonance angiography (MRA) were performed. DWI and FLAIR sequences revealed no abnormalities in the brain parenchyma (Figure 1 a-d) (DWI not shown). MRA did not provide good images because of motion artifacts. Therefore, we performed computed tomographic angiography (Figure 1 e), and no evident stenosis of the main artery was observed. However, there was slight stenosis of the left middle cerebral artery (MCA) M1 and right MCA M2 segments, which were thought to be flexure or mild sclerotic changes. Electroencephalography findings were normal.

We diagnosed the patient with MAS occurring in a TAWH, based on the ICH-III, Third Edition [1]. However, we also had to consider the possibility of RCVS due to mild stenosis of the bilateral MCA. Therefore, we prescribed verapamil hydrochloride (240 mg/day) owing to its vasodilating effect. We confirmed brain MRI and MRA findings 35 days after onset. Since no obvious changes were observed on brain MRI. MRA showed mild stenosis of the bilateral MCA as well as CTA findings. RCVS without headache is also rare, we assumed that RCVS could be ruled out. She had no recurrence of MAS. Therefore, we suspended verapamil hydrochloride. She experienced two visual auras 59 days after onset at 4:00 a.m. and 5:00 a.m., and another on the evening of 80 days after onset. As before, she experienced no other aura than visual aura, and no headache. The visual auras were as usual. We re-examined the brain MRI and MRA on day 84 of onset and found no changes. In the six-month follow-up period, there was no further recurrence of MAS.

## Discussion

In the present case, MAS occurred in a patient with only TAWH. She experienced only a visual aura and did not experience headaches or other auras. The diagnostic criteria for ICHD-III, Third Edition [1] of TWHA are as follows: A. Attacks fulfilling criteria for 1.2.1 migraine with typical aura and criterion B and B. No headache accompanies or follows the aura within 60 minutes [1]. When an aura occurs for the first time after the age of 40 years when symptoms are exclusively negative (e.g., hemianopia), or when the aura is prolonged or very short, other causes, particularly transient ischemic attacks, should be ruled out [1]. The prevalence of TAWH is 0.2%-6.5% [5]. Patients with TAWH only develop visual symptoms without headaches and often visit an ophthalmology clinic. In a retrospective study of Japanese ophthalmic clinics and university hospitals, 3.2% (35/1063) of the patients had TAWH [6].

The diagnostic criteria for MAS, mainly the frequency of aura onset and the number of days of continuous onset have been moderated from ICHD-II [2] to ICHD-III [1]. In the ICHD-II [2], the diagnostic criteria for MAS were experiencing two auras per day for at least five consecutive days [2]. In a study according to the ICHD-II [2], MAS was observed in four of 8,821 patients with migraine (0.05%) [7]. All MAS patients were female, with a mean age of 42.3 years (range: 29-63 years) and had MwA [7]. In ICHD-IIIβ (2013) [8], the diagnostic criteria were changed such that at least two auras occurred per day for three or more days. In a study according to the ICHD-IIIβ [8], 1.7% of patients had MAS in 477 cases of recurrent MwA [3]. In this study [3], 3.4% of the 477 cases of recurrent MwA had MAS according to the current ICHD-III criteria. Applying the ICHD-III, MAS patients were predominantly female (81.3%, 13/16), with a mean age of 54.50 years (range: 27-77 years), and occurred in MwA except for one case of TAWH [3]. MAS has been thought to be rare; however, it may have been overlooked, and its frequency may be higher than previously thought. In the literature as far as possible, as we investigated, most MAS reports have occurred in a typical MwA [3,4,7,9,10]. Additionally, 40-50% experienced only visual aura with MAS and 60-70% did not experience headache with MAS [3]. There has been one report of MAS with no consistent headache experience, as in the present case. A case report of a 74-year-old woman with TAWH complicated by MAS, with recurrent visual and speech aura during the onset of MAS, without headache [3]. The case we reported differed in that the patient experienced only visual aura. There have been cases of recurrence after 2-11 years, and long-term follow-up is necessary [3].

A typical migraine aura is a reversible focal neurological symptom that arises mainly before the onset of a migraine headache, and approximately one-third of migraine attacks are preceded by an aura [11]. A typical migraine aura may be a consequence of spreading cortical depolarization (CSD) in both animal models and humans [12]. The aura is characterized by visual, sensory, speech, motor, and “brain stem” disturbances [1]. The density of packing of the cells varies with the region of the cortex and is high in the visual area. The close proximity of the cells facilitates CSD, and visual symptoms are the most frequent in migraine auras [13]. Potassium facilitates the elicitation of CSD, and its clearance depends on glial cells. In humans, the lowest glial-to-neuronal cell ratio is observed in the primary visual cortex, where CSD is initiated occipitally [13]. Symptoms of an aura commonly develop gradually over 5-20 minutes and last for <60 minutes [1]. CSD may be involved in migraines, strokes, subarachnoid hemorrhages, and traumatic brain injuries [14]. Hence, an aura can occur in some diseases as well as in migraines [15-17]. In patients with aura, the following diseases that may provoke a circumscribed and prolonged increase in cortical excitability should be excluded: ischemic lesions, inflammatory diseases, migrainous infarction, epileptic disorders, neoplastic lesions, cerebral amyloid angiopathy, subdural hematoma, RCVS, and PRES [1,12,13,15]. In the medical interview, one of the main features that differentiate between the typical visual aura of migraine and other transient neurological conditions is the slow progression of symptoms in typical visual aura [15]. Visual seizures normally last for seconds and 1-3 minutes in some cases [12]. In addition, the visual change in a typical aura is not static but rather dynamic [13]. In addition to a detailed history and neurological findings, imaging and electroencephalography may be helpful in differentiating the disease.

This case report has limitations. The patient had difficulty with prolonged MRI. Cerebral amyloid angiopathy sometimes does not show abnormal findings on DWI and FLAIR sequences. However, T2*-weighted MRI and susceptibility-weighted images show microhemorrhages in some cases [18,19].

The pathogenesis of MAS is unclear. There is one literature report on two cases of MAS associated with a condition of hyperhomocysteinemia [9]. Additionally, three cases of MAS secondary to an organic brain lesion: a migrainous infarction, an acute ischemic stroke secondary to a vertebral artery dissection, and an inflammatory demyelinating disease of the central nervous system were reported [4]. Migraine is a common, disabling neurological disorder characterized by multiple phases, including premonitory, aura, headache, post-drome, and interictal [20]. It is commonly triggered by alterations in homeostasis, and the hypothalamus is considered a potential site of origin for migraine [20]. However, the role of the CSD in migraine onset remains unknown. The hypothesis that CSD is closely related to aura and activates the trigeminovascular system is strongly supported [21]. In addition, after calcitonin gene-related peptide infusion in 34 participants with MwA, 71% developed migraine headaches, and 38% developed migraine aura [22]. Because many migraineurs do not experience an aura, the hypothesis that CSD may also occur in subcortical regions in the form of “silent CSDs” and accordingly “silent auras” has been proposed [23]. However, demonstrating silent CSDs and auras is difficult. Another hypothesis is the parallel concept, which proposes that migraine aura and headache are separate events occurring in close temporal relations and in response to a shared initiating factor [22]. CSD most likely triggers a migraine aura, although it is expected that CSD is not always a sufficient trigger to develop a headache [13,22,23]. Although the mechanism is unknown in this case, lack of sleep, fatigue, and anxiety may have resulted in repeated CSDs, leading to complications of MAS.

There are few reports of treatment for MAS, and Haan et al. reported that in most of the patients of MAS, established migraine prophylaxis, such as sodium valproate and propranolol, was ineffective. In three of the patients, oral acetazolamide (two or three x 250 mg) was tried, and all reported improvement [10]. In this case, we did not use acetazolamide, in part because the patient did not experience a recurrence of MAS, and acetazolamide was not covered by insurance for migraine in Japan.

## Conclusions

MAS has been thought to be rare; however, it may have been overlooked, and its frequency may be higher than previously thought. The pathogenesis and effective treatment of MAS is unclear.

In addition to a detailed history, imaging is necessary in MAS cases to rule out organic brain disease. There have been cases of recurrence after 2-11 years, and long-term follow-up is necessary.
